# Supplementary material for: EnzML: multi-label prediction of enzyme classes using InterPro signatures
Source: BMC Bioinformatics. 2012 Apr 25;13:61. doi: 10.1186/1471-2105-13-61 (PMC3483700; doi:10.1186/1471-2105-13-61)
Supplement: Addtional file 5 — The Java code to format the data files, evaluate and predict. The file enzml_java_code.tar.gz contains the Java code used to format database data to ARFF and XML formats, to execute cross and train-test (jackknife) evaluations and to record evaluation results to database. More information is included in the readme.txt file and the Javadoc files. The code can be used with a MySQL database. To use a different database software, other JDBC drivers might be required. [file 1471-2105-13-61-S5.gz › java_code/enzml2011/doc/uk/ac/ed/inf/taweka/weka/DataSetManager.html]

DataSetManager


---


|  |  |  |
| --- | --- | --- |
| **Overview** \| **Package** \| **Class** \| **Use** \| **Tree** \| **Deprecated** \| **Index** \| **Help** | |  |
| **PREV CLASS**   **NEXT CLASS** | **FRAMES**    **NO FRAMES**     **All Classes** |  |
| SUMMARY: NESTED \| FIELD \| CONSTR \| METHOD | DETAIL: FIELD \| CONSTR \| METHOD |  |


---


## uk.ac.ed.inf.taweka.weka Class DataSetManager

```
java.lang.Object
  uk.ac.ed.inf.utils.database.Manager
      uk.ac.ed.inf.taweka.weka.DataSetManager
```

**Direct Known Subclasses:**: MulanDataSetManager

---

``` public class DataSetManager extends uk.ac.ed.inf.utils.database.Manager ```

DataSet generates a collection of Weka instances (rows of the ARFF file),
their attributes (column names) and their attribute values (cell values) The
last attribute is the class attribute.

**Version:**
:   20 Feb 2008

**Author:**
:   Luna De Ferrari luna.deferrari-at-ed.ac.uk

---

| **Field Summary** | |
| --- | --- |
| `static java.lang.String` | `DUMMY_VALUE` |


**Constructor Summary**

`DataSetManager(Arff generator)`


| **Method Summary** | |
| --- | --- |
| `Arff` | `getArffGenerator()` |
| `ArffProperties` | `getArffProperties()` |
| `java.lang.String` | `getClassName()` |
| `DataSetDbLoader` | `getDataLoader()` |
| `DataSetChecker` | `getDataSetChecker()` |
| `DataSetGenerator` | `getDataSetGenerator()` |
| `java.lang.String` | `getDataSetName()` |
| `DataSetWriter` | `getDataSetWriter()` |
| `boolean` | `initialisationIsCorrect()` |
| `void` | `writeArff()` |

**Methods inherited from class java.lang.Object**

`equals, getClass, hashCode, notify, notifyAll, toString, wait, wait, wait`

**Field Detail**

### DUMMY\_VALUE

```
public static final java.lang.String DUMMY_VALUE
```

**See Also:**: Constant Field Values


**Constructor Detail**

### DataSetManager

```
public DataSetManager(Arff generator)
```

**Parameters:**: `generator` - a generator object to write this dataset to file


**Method Detail**

### getArffGenerator

```
public Arff getArffGenerator()
```

---


### getArffProperties

```
public ArffProperties getArffProperties()
```

---


### getClassName

```
public java.lang.String getClassName()
```

---


### getDataLoader

```
public DataSetDbLoader getDataLoader()
```

---


### getDataSetChecker

```
public DataSetChecker getDataSetChecker()
```

---


### getDataSetGenerator

```
public DataSetGenerator getDataSetGenerator()
```

---


### getDataSetName

```
public java.lang.String getDataSetName()
```

---


### getDataSetWriter

```
public DataSetWriter getDataSetWriter()
```

---


### initialisationIsCorrect

```
public boolean initialisationIsCorrect()
```

---


### writeArff

```
public void writeArff()
```


---


|  |  |  |
| --- | --- | --- |
| **Overview** \| **Package** \| **Class** \| **Use** \| **Tree** \| **Deprecated** \| **Index** \| **Help** | |  |
| **PREV CLASS**   **NEXT CLASS** | **FRAMES**    **NO FRAMES**     **All Classes** |  |
| SUMMARY: NESTED \| FIELD \| CONSTR \| METHOD | DETAIL: FIELD \| CONSTR \| METHOD |  |


---
